# Neuroprotective Effect of Pentoxifylline on 3,4-Methylenedioxymethamphetamine-Induced Apoptosis in CA1 Cells of Wistar Rat Hippocampus

**DOI:** 10.31661/gmj.v8i0.963

**Published:** 2019-08-07

**Authors:** Shabnam Movassaghi, Zeinab Khazaei Koohpar, Mehrdad Hashemi, Sourena Jafari Semnani, Zahra Nadia Sharifi

**Affiliations:** ^1^Department of Anatomical Sciences and Cognitive Neuroscience, Faculty of Medicine, Tehran Medical Sciences, Islamic Azad University, Tehran, Iran; ^2^Department of Cellular and Molecular Biology, Faculty of Biological Sciences, Tonekabon Branch, Islamic Azad University, Tonekabon, Iran; ^3^Department of Genetics, Faculty of Advanced Sciences and Technology, Islamic Azad University, Tehran Medical Sciences, Tehran, Iran; ^4^Faculty of Pharmacy, Tehran University of Medical Sciences, Tehran, Iran

**Keywords:** Pentoxifylline, 3,4-Methylenedioxymethamphetamine, Apoptosis, Hippocampus

## Abstract

**Background:**

3,4-Methylenedioxymethamphetamine is psychoactive and hallucinogenic and has been shown to produce neurotoxicity both in animals and in humans. Recently, vasodilator drugs such as pentoxifylline (PTX) have been introduced as an alternative with neuroprotective effects. There is no study about the protective effect of PTX on hippocampal apoptosis due to high-dose administration of 3,4-Methylenedioxymethamphetamine (MDMA), so in this study, the protective effect of PTX on the hippocampus of male Wistar rats following high-dose of the drug has been investigated.

**Materials and Methods:**

Twenty-four male Wistar rats weighing 250-300 g were randomly divided into four groups: control, sham (MDMA injection), experimental (MDMA+PTX injection), and vehicle (MDMA+saline) groups. Two weeks later, the brains were removed and prepared for TUNEL and western blot techniques. Concomitantly the hippocampus was removed to study the change in *Bcl-2* and *BAX* mRNA expression with quantitative real-time polymerase chain reaction.

**Results:**

Data showed that the number of apoptotic bodies significantly decreased in the experimental group compared to the other groups, except for in control. Also, further investigation revealed that *BAX* reduced considerably, while *Bcl-2* mRNA expression increased dramatically after PTX treatment.

**Conclusions:**

Our results suggest that PTX may be a neuroprotective agent, and its neuroprotective potential may contribute to reducing the severity of lesions in the hippocampus following a high dose administration of MDMA.

## Introduction

The 3, 4-Methylenedioxymethamphetamine (MDMA), also known as ecstasy, is a psychoactive substance used as a recreational drug [1, 2]. MDMA can damage the serotonergic system by affecting a pruning of serotonergic fibers and terminals of forebrain target areas such as the neocortex, hippocampus, and striatum [3]. This damage is well-known in laboratory animals, and, it may also occur in persons who are heavy users [4]. Several factors contribute to the neurotoxicity induced by MDMA such as hyperthermia, metabolism of dopamine and serotonin, nitric oxide, glutamate-induced excitotoxicity, and serotonin 2A receptor agonist [1]. Amphetamine and amphetamine derivatives induce apoptosis upon acute and repeated exposures. Many studies indicated that MDMA could induce apoptosis in cortex and cerebellum [2, 5, 6]. It seems that apoptosis pathways induced by methamphetamine can occur via caspase-independent mechanisms by the mitochondrial apoptotic pathway, associated with a *Bcl-2* levels reduction [7]. Apoptosis of neurons characterized by DNA cleavage and differential expression of antiapoptotic and proapoptotic proteins (BCL-XL/S variants) accompanied by the activation of caspase-3 in neuronal cultures has been shown in some researches. Caspase-3, a member of the caspases family, is involved in the process of the apoptotic cascade (together with caspase-2 and -7). Part of this study focused on this specific enzymatic activity to show its important role in apoptosis in CA1 cells of rat hippocampus [8].Pentoxifylline (PTX) is a phosphodiesterase inhibitor used to treat muscle pain in peripheral vascular diseases. The effects of this drug seem to be related to alterations in cellular functions and improvement of microcirculatory perfusion in both peripheral and cerebral vessels [9, 10]. Many experiments indicated additional therapeutic anti-inflammatory and immunomodulatory effects of PTX [11-13]. The study aimed to determine the effect of PTX administration as a neuroprotective on hippocampal apoptosis due to the administration of MDMA. Also, the effects of PTX on MDMA-induced alterations in the hippocampus *BAX* and *Bcl-2* expressions in rats were evaluated.

## Materials and Methods

### Animals and Animal Treatment

Twenty-four young adult, male Wistar rats (age: 3- 4 months, body weight: 250-300 g) were acquired from the pharmacology department of the Tehran University of Medical Sciences. The rats were housed at a controlled temperature (22±1°C) on a 12 h alternating light/dark cycle. Food and water were provided ad libitum. The experimental procedures performed to adhere to the ethical principles set down by the ethics committee for the use of experimental animals at Islamic Azad University, Tehran Medical Branch (approval code: 15758). All materials were purchased from Sigma (Germany) except MDMA, which was synthesized at the

medical chemistry lab of the Tehran University of Medical Sciences and PTX, which gifted by the Amin Pharmaceutical Co (Isfahan,-Iran). Animals were randomly allocated into four different groups:

1. Control: rats kept in the animal house for two weeks.
2. Sham: rats received an intraperitoneal (IP) injection of MDMA every 2h (7.5 mg/kg).
3. Experimental: rats were given 200 mg/kg PTX [14] and after one week, received MDMA every 2h (7.5 mg/kg).
4. Vehicle: rats received an IP injection of MDMA (7.5 mg/kg, × 3, 2 h interval) and after one week, normal saline (vehicle of PTX) was injected.

Animals were euthanized two weeks (after all the experiments started). Then, half of their brains were removed and post-fixed in the fixator (paraformaldehyde 4%) for more than three days, and the other half of each brain was immediately placed on a dish standing on crushed ice to dissect the hippocampus. The selected area was snap frozen in liquid nitrogen and kept at -80°C.

### Detection of Apoptosis by TUNEL Staining

TUNEL staining was performed using an In Situ Cell Death Detection Kit (Roche, Mannheim, Germany) to detect apoptotic cells. Sections were routinely deparaffinized and hydrated. After washing in phosphate-buffered saline (PBS) and deproteinized by proteinase K (20 *µ*g/ml) for 30 min, sections were rinsed in 3% H2O2 in methanol for 10 min to inactivate the endogenous peroxidase. After incubating in the TUNEL reaction mixture for 60 min, the sections were rinsed with PBS and visualized by using Converter-POD for 30 min in the dark, and then rinsed with PBS after adding 50–100 *µ*L 3,3-Diaminobenzidine mounted by coverslip and analyzed by light microscope. Five fields were randomly selected from each section at high magnification (×400), and the number of TUNEL positive cells in the CA1 region was counted and averaged for statistical analysis. All counting procedures were performed blindly.

### Caspase-3 Activity

Fresh hippocampus was homogenized in an ice-cold lysis buffer that contained tris HCl (50 mM, pH 8.0), NaCl (150 mM), Nonidet P-40 (1%), glycerol (10%), phenylmethylsulfonyl fluoride (10 ml/ml), sodium deoxycholate (0.5%), and aprotinin (30 ml/ml), in addition to a protease inhibitor cocktail (Roche Applied Science, Germany). The homogenized hippocampus was subjected to centrifugation at 12000 g for 20 min at 4oC, and the supernatant was collected. 100 µg from the total protein of the supernatant was loaded onto each lane and electrophoresed on SDS-PAGE gels (10%). Proteins were transferred onto nitrocellulose membranes for 1 h at room temperature and blocked with PBS that contained non-fat dried milk powder (5%) for 2h. Membranes were washed by tris buffer that contained Tween 20 (1%), then probed with a monoclonal anti-cas3 antibody (1:1000; Abcam, St. Louis, MO, USA) overnight, after which a secondary anti-rabbit akp-linked antibody (1:10000; Abcam,) was added for 1 h at room temperature, then the membranes were stained with BCIP/NBT. The β-actin served as a positive control for protein loading, and a standard high range molecular weight was used to determine protein sizes. Results were evaluated by the UVIdoc program (UVIdoc version 12.6 for Windows, copyright 2004).

### RNA Isolation and Real-time Polymerase Chain Reaction (PCR)

Total RNA was extracted from 100 mg of frozen hippocampus tissue using RNA isolation reagent (Sigma, Germany) as recommended by the manufacturer, the extracted RNA was purified using the RNeasy Mini Kit (Qiagen Hilden, Germany), and cDNA was synthesized using Quantitect Reverse Transcription Kit (Qiagen Hilden, Germany) according to the manufacturer’s instructions. Reverse transcription was carried out as follows:

42˚C for 2 min, 42˚C for 15 min, and 95˚C for 3 min (one cycle). cDNA was stored at -20ºC for PCR. Real-time PCR was performed in a 25 µl of the reaction solution. The following sequences were used as primers: *Bcl-2* forward primer, 5´ATCGCTCTGTGGATGACTGAGTAC3´, and *Bcl-2* Reverse primer, 5´AGAGACAGCCAGGAGAAATCAAAC3´, *BAX* forward primer, 5´GGGTGGCTGGGAAGGC3´, and *BAX* reverse primer, 5´TGAGCGAGGCGGTGAGG3´, GAPDH forward primer, 5´AAGTTCAACGGCACAGTCAAGG3´, and GAPDH reverse primer, 5´ CATACTCAGCACCAGCATCACC3´. Real-time PCR carried out in an optical grade 96-well plates (Micro amp, Applied Biosystems, Singapore) at a reaction volume of 25 µl, including 12.5 SYBR Green Master Mix (Primer Design), 300 nm primer and 5ng template DNA. All samples were run in duplicate. Thermal cycling was performed on the Applied Biosystems 7300 real-time PCR system. Threshold cycle (Ct) data were collected using the ABI Prism 7300 Sequence Detection System version 1,2,3 (Applied Biosystems, UK). The relative quantification of gene expression was analyzed by the 2- ΔΔCt method. The fold change in target gene cDNA relative to the GAPDH internal control was determined by:

fold change=2^-ΔΔCt^, where ΔΔCt=(Ct_target gene_- Ct_GAPDH_)-(Ct_control_-Ct_GAPDH_).

### Statistical Analysis

Results were expressed as mean±standard deviation. The one-way analysis of variance (ANOVA) was used to evaluate the difference between multiple groups followed by the post hoc test (Tukey). The data were analyzed by SPSS version 19.0 software (SPSS, Inc., Chicago, Illinois, USA), and P<0.05 was assessed as statistically significant.

## Results

**Figure 1:**
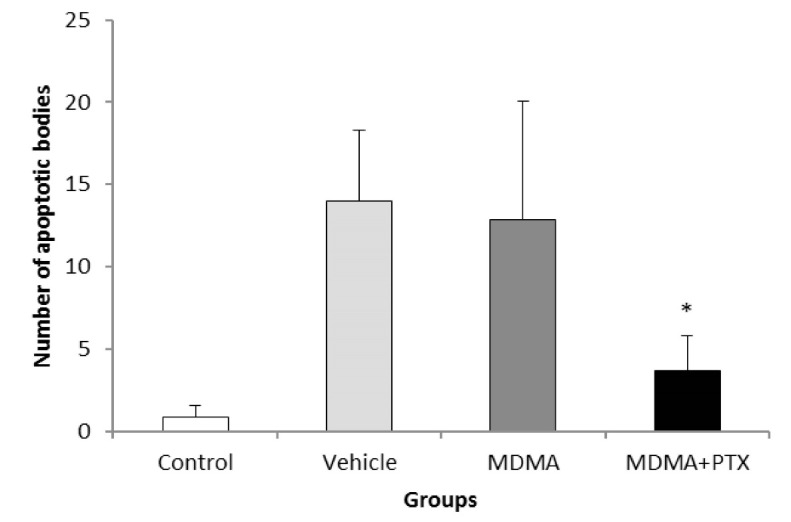
Comparison of the mean number of apoptotic bodies in the vehicle, experimental (PTX+MDMA), MDMA, and control groups according to TUNEL assay. The mean number of apoptotic bodies significantly decreased in the hippocampus of the experimental group following PTX (200 mg/kg) treatment in comparison with other groups. However, there was no statistical difference between the number of apoptotic bodies in the control and experimental groups. Data are presented as means± SD. *P<0.05 vs. MDMA and vehicle groups

**Figure 2:**
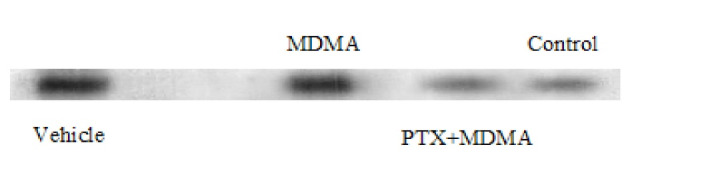
Effect of PTX on MDMA induced cell apoptosis. The western blotting analysis indicated that the PTX treatment could decrease the expression of caspase-3 following high dose administration of MDMA.

**Figure 3:**
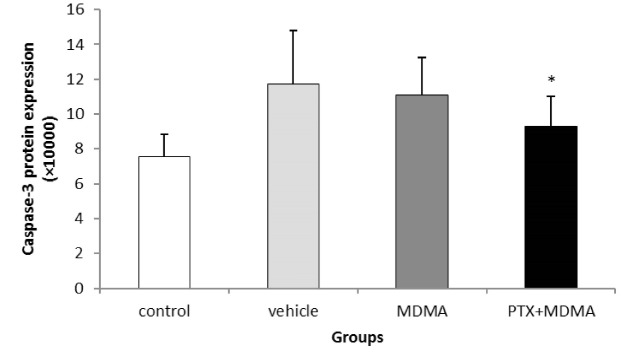
Protein expression of cleaved caspase-3 in the hippocampus of the vehicle, experimental (PTX+MDMA), MDMA, and control groups by western blot. Expression of caspase-3 protein significantly decreased in the hippocampus of the experimental group following PTX (200 mg/kg) treatment. Data are presented as means±SD. *P<0.05 vs. MDMA and vehicle groups

**Figure 4:**
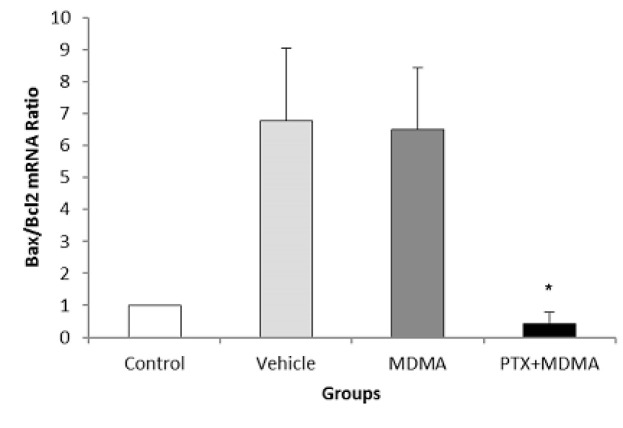
*BAX*/*Bcl-2* mRNA ratios in the hippocampus of rats. Data are presented as means± SD. *P<0.05 vs. MDMA and vehicle groups

Data that were collected from the TUNEL staining method based on DNA damage-induced apoptosis showed that the number of apoptotic bodies increased in the CA1 region of the hippocampus after high dosage of MDMA, and there was a significant difference between the control versus MDMA groups (P=0.005). No statistically significant difference was seen between the control group and the experimental group, which received PTX before using MDMA (P=0.881, Figure-1). Results from western blot analysis showed that expressions of activated caspase-3 were markedly increased in the hippocampus due to MDMA administration compared with the control group. PTX treatment could decrease the expression of caspase-3 and apoptosis in the hippocampus. Thus, the protein expression of activated caspase-3 in the hippocampus of the experimental group that was treated with PTX was similar to that in the hippocampus of the control group (Figure-2 and 3). The results also showed that *Bcl-2* and *BAX* mRNA levels were decreased and increased respectively in the hippocampus due to MDMA administration compared with the control group, and there was a significant difference between the control versus MDMA groups. No statistically significant difference was seen between the control and experimental group that was treated with PTX. Also, we found that MDMA caused a considerable increase in the pro-death/anti-death mRNA ratios for *BAX*/*Bcl-2* compared with the control. On the other hand, the real-time PCR result revealed that PTX treatment significantly decreased *BAX* mRNA levels in MDMA-injected rats, and increased *Bcl-2* mRNA levels). Also, PTX treatment significantly reduced the ratio of *BAX*/*Bcl-2* compared to the MDMA group (Figure-4).

## Discussion

Our data showed that a high dose administration of MDMA has a neurotoxic effect on the CA1 region of the hippocampus with a stimulating apoptotic pathway. Methamphetamine and its derivatives are potent psychostimulants that increase the glutamate level, which is neurotoxic in high concentrations, and the mechanism of action is possibly due to the oxidative stress caused by reactive oxygen species generation in the mammalian brain [15]. Furthermore, serotonergic and dopaminergic neuronal cell damage has been observed in experimental amphetamine intoxication in laboratory animals [2]. Amphetamine and amphetamine derivatives induce apoptosis following acute and repeated exposures. Apoptotic pathways induced by amphetamine and methamphetamine in neurons seem to be mainly mediated by the mitochondrial apoptotic pathway associated with a decrease in *Bcl-2* levels and direct interference with mitochondrial transmembrane potential [9]. Methamphetamine induces the significant increase in the pro-death *Bcl-2* family genes *Bad*, *BAX*, *Bid,* and decreases in the anti-death genes *Bcl-2* and Bcl-XL [16, 17]. Our data showed that seven days after systemic administration of MDMA (7.5 mg/kg, IP, × 3, 2 h apart) the caspase-3 activity significantly increased in hippocampus Caspases are a family of proteases enzymes that are essential in programmed cell death, development and inflammation. Sequential activation of caspases plays a central role in the execution-phase of cell apoptosis. The caspase-3 protein is a member of the cysteine-aspartic acid protease (caspase) family. It is a caspase protein that is encoded by the caspase-3 gene and interacts with caspase-8 and -9. Caspase-3 protein cleaves and activates caspases-6 and -7; and the protein itself is processed and activated by caspases-8, 9, and 10. It is the dominant caspase involved in the cleavage of amyloid-beta 4A precursor protein, which is associated with neuronal death in neurodegenerative diseases [18]. This study showed that MDMA administration increases caspase-3 activity in selected brain area such as the hippocampus, which is supported by Capela who is suggested that direct MDMA 5-HT (2A)-receptor stimulation produces intracellular oxidative stress that leads to neuronal apoptosis in the brain cortex via caspase-3 activation [19]. We have recently shown that MDMA administration significantly decreased Bcl-2 mRNA levels [20]. Our investigation revealed that a toxic regimen of MDMA caused significant increases in the pro-death *Bcl-2* family gene expression *BAX*. Besides, we found that MDMA caused significant increases in the pro-death/anti-death mRNA ratios for *BAX*/*Bcl-2*. *Bcl-2* inhibits cytochrome C release from mitochondria elicited by pro-apoptotic *BAX*, resulting in the inhibition of caspase activation and apoptotic death [21]. Our results were in agreement with the results reported by Jayanthi *et al.* [17]. PTX is a methylxanthine well-known for its roles as a phosphodiesterase inhibitor, a ligand of adenosine receptors, a modulator of the ryanodine Ca2+-release channel of the endoplasmic reticulum, and a downregulation of TNF-α [22, 23]. Some in vitro and in vivo experiments indicated a therapeutic potential for PTX as an anti-inflammatory agent. This drug can inhibit the production of TNF-alpha, as well as other pro-inflammatory cytokines [24]. An earlier study [25] indicated that TNF-alpha and glutamate could act synergistically to induce neuronal cell death. Recently, many anti-inflammatory properties of PTX have been described, including the decrease of inflammatory mediator production and the prevention of neuronal apoptosis, and decreased caspase-3 activity [26]. The study data indicate that the number of apoptotic bodies significantly decreased with PTX treatment. It appears that the neuroprotective effect of PTX can reduce the severity of lesions and apoptosis in the hippocampus following a high dose administration of MDMA. We have recently shown that PTX treatment up-regulated *Bcl-2* in the hippocampus of MDMA-injected rats [20]. *Bcl-2* has been reported to protect against generators of reactive oxygen species, to increase antioxidant defenses, and to decrease levels of reactive oxygen species and oxidative damage [27]. In the present study, *BAX* mRNA expression in hippocampus tissues was significantly decreased when rats were treated with PTX. In addition, we found that PTX treatment leads to significant decreases in the pro-death/anti-death mRNA ratio for *BAX*/*Bcl2*. Cell survival is thought to depend critically on a molecular balancing act that regulates the ratios of *Bcl-2* to *BAX*, *BAD* to *BCL-XL*, or *Bcl-2*. Because apoptosis is thought to result from changes in these ratios, with the consequent release of cytochrome C and the activation of effector caspases [17]. Therefore, the effect of PTX in preventing the MDMA-induced increase of *BAX*/*Bcl-2* ratio may be associated with its protective role on apoptosis in the hippocampus. In the present study, we have shown that the administration of PTX is associated with preventing MDMA induced increase in the pro-death/anti-death ratio for *BAX*/*Bcl-2*, suggesting a mechanism for the observed neuroprotection in the MDMA-induced neurotoxicity model. Therefore, it seems that PTX can prevent the inflammatory response and reduce the neural toxicity induced by MDMA by decreasing the activation of caspase-3.

## Acknowledgment

We would like to thank the Amin Pharmaceutical Co (Isfahan, Iran) for their generous gift of PTX. This research project has been financially supported by the office of the vice chancellor for research, Tehran Medical Branch, Islamic Azad University (grant number: 15758). We would also like to thank the Medical Science Research Center, Tehran Medical Branch, Islamic Azad University, Tehran, Iran, for supporting this project.

## Conflict of Interest

The authors declare no conflicts of interest and have approved the final article.
